# Can Classification and Regression Tree Analysis Help Identify Clinically Meaningful Risk Groups for Hip Fracture Prediction in Older American Men (The MrOS Cohort Study)?

**DOI:** 10.1002/jbm4.10207

**Published:** 2019-08-21

**Authors:** Yi Su, Timothy C Y Kwok, Steven R Cummings, Benjamin H K Yip, Peggy M Cawthon

**Affiliations:** ^1^ Department of Medicine and Therapeutics, Prince of Wales Hospital The Chinese University of Hong Kong Shatin Hong Kong China; ^2^ Jockey Club Centre for Osteoporosis Care and Control The Chinese University of Hong Kong Shatin Hong Kong China; ^3^ California Pacific Medical Center Research Institute San Francisco CA USA; ^4^ University of California, San Francisco San Francisco CA USA; ^5^ Jockey Club School of Public Health and Primary Care, The Chinese University of Hong Kong Shatin Hong Kong China

**Keywords:** AGE, BMD, CART, HIP FRACTURES, RISK PREDICTION

## Abstract

Although the WHO fracture risk algorithm (FRAX) is used to predict fracture, the utility of some simple machine‐learning methods, such as classification and regression trees (CARTs) should be evaluated to determine their efficacy in fracture prediction. Follow‐up time for the hip fracture analyses of 5977 community‐dwelling American men aged ≥65 years old was truncated to 10 years. There were 172 (2.9%) men who had an incident nontraumatic hip fracture. The CARTs were developed using hip BMD and common clinical risk factors as follows: model 1 = using classification with continuous variables of age, total hip BMD, and femoral neck BMD, or together with common clinical risk factors; and model 2 = using classification with continuous variables of age, total hip BMD, femoral neck BMD, FRAX score, osteoporosis by *T*‐score at the hip, and common clinical risk factors. The predictive performance of risk models derived from CARTs was compared with the basic classification of FRAX at 3% (basic model). From model 1, discriminators selected by CART were total hip BMD, age, and femoral neck BMD; no other clinical risk factors were selected. From model 2, discriminators selected by CART were FRAX score, femoral neck BMD, and age. Compared with the basic model using only a high‐risk group by FRAX ≥3%, no significantly improved predictive performance was demonstrated by model 1 or model 2 as identified by CART with the area under the receiver‐operating characteristic curve for each model of 0.714 (95% CI, 0.676 to 0.751) or 0.726 (95% CI, 0.690 to 0.762) versus 0.703 (95% CI, 0.667 to 0.740), respectively. The improved overall net reclassification improvement index was 0.02 (95% CI, –0.04 to 0.08) and 0.05 (95% CI, –0.01 to 0.10), respectively. Although a FRAX category is a good clinical indicator for hip fracture risk, a simple classification by age and BMD may provide an alternative way to estimate a clinical risk level of 3.0%. © 2019 The Authors. *JBMR Plus* is published by Wiley Periodicals, Inc. on behalf of the American Society for Bone and Mineral Research.

## Introduction

Hip fractures seriously threaten the health of older people. The corresponding global medical and social burdens of hip fracture increase in concert with our aging population.^1^ Accurate and individualized hip fracture risk prediction may guide an effective preventive intervention that would reduce the incidence of hip fracture and relieve its burden. Many previous studies have attempted to quantify hip fracture risk; today, the most widely used risk assessment tools are the level of osteoporosis based on BMD *T*‐scores^2^, ^3^ and the WHO fracture risk algorithm (FRAX).^4^, ^5^

The diagnostic criteria for osteoporosis defined as a *T*‐score threshold of –2.5 in BMD was first established for women, initially intended primarily for descriptive epidemiology and subsequently proposed as an intervention threshold aiming for fracture prevention.^3^ However, the threshold was arbitrarily defined, and most fragility fractures occur in individuals with a BMD value above the operational threshold for osteoporosis.^6^, ^7^ FRAX was subsequently developed to determine absolute fracture risk to enable better fracture prediction than a BMD *T*‐score alone.^4^ Currently, the cost‐effective thresholds of absolute hip fracture risk by FRAX are applied for clinical assessment and intervention.^5^, ^8^, ^9^, ^10^ However, the identified genetic determinants of fracture in a recent meta‐analysis of genome‐wide association studies (GWASs) were all related to BMD, and among the common clinical risk factors for fracture, only BMD showed a major causal effect on fracture.^11^ Further is needed to develop a more‐effective clinical decision rule.

As an automatic “machine‐learning” method, classification and regression tree (CART) analysis^12^ can be used to classify people into ordinal risk or clinically important categories. This tree‐building technique could help in the selection of risk factors irrespective of their distributions or interactions^13^; it has been shown to be effective in the development of clinical decision rules that perform as well or better than rules developed using more traditional methods.^14^, ^15^, ^16^, ^17^, ^18^ This simple classification tool may also help to provide a reference pattern of risk discrimination for clinical decision‐making on hip fracture prevention.

Thus, we aimed to describe alternative risk classifications using CART analysis for hip fracture prediction in a community‐based cohort of 5994 older American men: the Osteoporotic Fractures in Men (MrOS) Study. And we further compared its predictive performance with FRAX in the clinical context of the United States.

## Materials and Methods

### Study participants

The Osteoporotic Fractures in Men (MrOS) Study is a prospective cohort study designed to identify the determinants of fracture in men (http://mrosdata.sfcc‐cpmc.net). Design and recruitment have been previously described.^19^, ^20^ From March 2000 to April 2002, 5994 community‐dwelling men 65 years of age or above were recruited at six clinical sites in the United States (Birmingham, AL; Minneapolis, MN; Palo Alto, CA; Pittsburgh, PA; Portland, OR; San Diego, CA). Written informed consent was obtained for all participants. The institutional review board at each clinical site approved the study.

### Data collection and measurements

Men completed a standardized questionnaire and interview, which included items about demographics, medical history, fracture history, parental fracture history, medication, smoking status, and alcohol consumption. Body height was measured on a Harpenden stadiometer (Seritex, DyFed, Wales) and weight was measured on balance beam scales (except the MrOS Portland site, which used a digital scale) that were calibrated with standard weights. BMI was calculated as weight (kg)/height (m^2^).

BMD (g/cm^2^) was measured in the proximal femur and lumbar spine using DXA measured by Hologic QDR 4500 densitometers (Hologic, Waltham, MA, USA) during the baseline visit. The densitometers across study centers were cross‐calibrated with a standard spine phantom to ensure consistency and quality of bone mass measurement. Osteoporosis was defined using either the femoral neck BMD *T*‐score or the total hip BMD *T*‐score ≤–2.5, which was calculated based on the NHANES III (Third National Health and Nutrition Examination Survey) reference database for femoral neck and total hip measurements in women aged 20 to 29 years.^21^

The US‐FRAX (version 3.12) 10‐year risk estimate of hip fracture was calculated at the WHO Collaborating Centre for Metabolic Bone Disease, University of Sheffield, UK. Data on age, BMI, and race are required for FRAX score calculation. If data were missing on any of the other clinical risk factors in the FRAX, a “null” response for the corresponding value was assumed and a risk was calculated without that information. The risk threshold for a 10‐year hip fracture probability of 3.0% was used referring to its cost‐effective treatment threshold in the United States.^5^ The FRAX category was defined as low risk for those below the threshold and high risk for those equal to or above the threshold.

Participants brought all medications into the clinical center that they had taken within the previous 30 days; all medications were then coded.^22^ If a participant forgot to bring his medications, clinic staff obtained this information over the telephone or at a return visit. Medications were entered into an electronic database and were matched to their ingredient(s) on the basis of the Iowa Drug Information Service Drug Vocabulary (College of Pharmacy, University of Iowa, Iowa City, IA, USA).^23^ Sensitivity analyses were conducted by excluding participants with bisphosphonate use before the baseline visit or during the follow‐up. Bisphosphonate use was defined as any bisphosphonate reported to be taken within 30 days prior to any visit.

### Hip fracture

After the baseline assessments, a questionnaire was mailed to participants every 4 months to ascertain incident fractures. Follow‐up for fractures and vital status exceeded 99% completion. All reported fractures were validated by centralized physician review of radiology reports or X‐rays if no radiology report was available. Follow‐up time for the hip fracture analyses was truncated to 10 years to correspond with the 10‐year fracture probability estimates from FRAX. An expert panel rated fractures as associated with “severe” trauma if they occurred during a motor vehicle accident or trauma equivalent to falling from more than one stair‐step above standing height. The analyses included all first nontraumatic hip fractures as the endpoints.

### Statistical analysis

The differences in baseline characteristics between hip fracture and nonfracture were compared using the Student's *t*‐test for continuous variables, and Pearson chi‐square test for categorical variables.

The main CARTs were developed as follows. Model 1 was a simple model using the continuous variables of age, total hip BMD, and femoral neck BMD. We also ran model 1a that included common clinical risk factors (including BMI, height, weight, history of fracture, parental history of hip fracture, smoking, alcohol consumption, use of corticosteroids, prevalence of rheumatoid arthritis, presence of secondary osteoporosis, and fall history in the previous one year). Model 2 was a more complex model using the continuous variables of age, total hip BMD, femoral neck BMD, FRAX score, osteoporosis status (by *T*‐score at the hip), and common clinical risk factors as mentioned above. In addition, we ran two secondary CART models: model 3, which used common clinical risk factors (that is, excluding BMD) in a simple model (eg, model 1), and model 4, which used a dichotomous variable of FRAX score (≥3.0%) rather than the continuous FRAX score in model 2. For all models, the number of splits and complexity parameters was controlled to prevent overfitting and guarantee a more parsimonious model tree that used a fixed complexity parameter (C_p_ = 0.01). Internal validation was conducted by 10‐fold cross‐validation.

We used Cox proportional hazard models to evaluate the association between newly identified risk groups separate for those factors identified from CART models 1, 1a, 2, 3, and 4, and the risk of subsequent hip fracture. The predictive performances of the newly identified classification models were compared with the traditional identification: a very basic model (model 0, which included only a FRAX score above or below 3.0% as the only predictor). Predictive performance was evaluated by sensitivity, specificity, the area under the receiver‐operating characteristic curve (AUC was calculated from the logistic regression model) and the net reclassification improvement index (NRI).^24^

All statistical tests were two‐tailed with *P* < 0.05 considered significant. Statistical analyses were performed using SAS 9.4 (SAS Institute, Cary, NC, USA) and R software (version 3.4.0; R Foundation for Statistical Computing, Vienna, Austria).

## Results

Of the 5994 men, 5977 (99.7%) had follow‐up for hip fracture. In 10 years of follow‐up, 172 (2.9%) men had an incident nontraumatic hip fracture, 1598 (26.7%) had died, and 210 (3.5%) terminated the program. The average follow‐up period was 8.6 ± 2.5 years. The men predominately were white (89.4%), and had a mean age of 73.6 ± 5.9 years and an average BMI of 27.4 ± 3.8 kg/m^2^. Compared with those men without an incident hip fracture, those with a hip fracture were older, had lower BMI and lower BMD, were more likely to report current smoking, and to have had a prior fracture or fall history. The men with an incident hip fracture were more likely to have been osteoporotic (femoral neck or total hip BMD *T*‐score ≤–2.5) or be categorized into the hip fracture high‐risk group by FRAX at baseline (Table 1).

**Table 1:** Baseline Characteristics of Hip Fracture and Nonhip Fracture Subjects With a 10‐Year Follow‐Up

Characteristics	Nonhip fracture, Mean ± SD/*N* (%)	Hip fracture, Mean ± SD/*N* (%)	*P* value
Age (year)	73.5 ± 5.8	78.0 ± 6.1	<0.001
BMI (kg/m^2^)	27.4 ± 3.8	26.4 ± 3.8	0.001
Total hip BMD (g/cm^2^)	0.96 ± 0.14	0.82 ± 0.13	<0.001
Femoral neck BMD (g/cm^2^)	0.79 ± 0.13	0.67 ± 0.11	<0.001
Race			0.041
Asian	190 (3.3)	1 (0.6)	
African American	241 (4.2)	2 (1.2)	
White or other	5252 (90.5)	166 (96.5)	
Hispanic	122 (2.1)	3 (1.7)	
Previous fracture = 1	1287 (22.2)	64 (37.2)	<0.001
Parental history of hip fracture = 1	740 (12.7)	18 (10.5)	0.441
Use of corticosteroids = 1	123 (2.1)	2 (1.2)	0.553
Current smoking = 1	194 (3.3)	12 (7.0)	0.018
Rheumatoid arthritis = 1	301 (5.2)	14 (8.1)	0.125
Alcohol use = 1	232 (4.0)	5 (2.9)	0.601
Fall history in the previous year = 1	1208 (20.8)	56 (32.6)	<0.001
Osteoporosis^a^ = 1	117 (2.0)	30 (17.4)	<0.001
High‐risk category of^b^ FRAX = 1	2299 (39.6)	119 (69.2)	<0.001
High‐risk category^b^ of FRAX (BMD) = 1	1249 (21.5)	107 (62.2)	<0.001
Total number	5805 (97.1)	172 (2.9)	—

^a^ Osteoporosis defined as femoral neck/total hip BMD *T*‐score ≤–2.5.

^b^ High‐risk category defined as the FRAX score (including BMD or not) at the threshold of 3%.

“1” indicates a yes response.

FRAX = fracture risk assessment tool.

**Figure 1:**
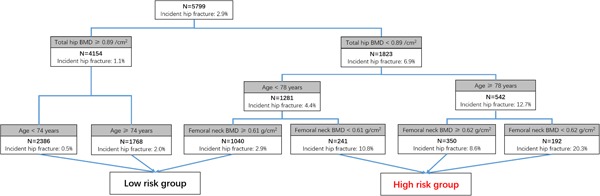
The structured risk tree for hip fracture prediction developed by CART (classification and regression tree) analysis using hip BMD and age—from model 1.

In the simple model (model 1), the most important predictors selected by CART were total hip BMD, age, and femoral neck BMD. The absolute cross‐validated error rate (= root node error × cross‐validated error rate) for 10‐group internal validation was approximately 25%. Figure 1 shows the simple model (model 1). The first node was above or below total hip BMD of 0.89 g/cm^2^. For those below this value, there were two additional nodes: age above or below 78 years and femoral neck BMD above or below 0.61 g/cm^2^. Three subgroups were identified as high risk according to their 10‐year hip fracture incidence rate (≥3.0%). Therefore, 783 (13.1%) men were identified as high risk for hip fracture; 95 (12.1%) of whom had an incident hip fracture during the 10‐year follow‐up. There were 5194 (86.9%) men identified as low risk for hip fracture; 77 (1.5%) of whom had an incident hip fracture. Compared with the low‐risk group, the HR of the high‐risk group was 9.95 (95% CI, 7.36 to 13.44; see Table 2). When total hip and femoral neck BMD were excluded in the secondary analysis (model 3), age was identified as the only predictor with clinical meaning at the risk of 3.0%.

**Table 2:** The Comparisons of Predictive Abilities Among Different Risk‐Identified Methods for Hip Fracture

	Risk group classification (by 2 groups)					
	Basic model (model 0)		Simple model by CART (model 1)		Complex model by CART (model 2)	
Index	HR	95% CI	HR	95% CI	HR	95% CI
High‐/low‐risk group	6.46	4.74 to 8.79	9.95	7.36 to 13.44	8.60	6.29 to 11.76
Sensitivity	0.62	0.55 to 0.69	0.55	0.47 to 0.62	0.65	0.57 to 0.72
Specificity	0.78	0.77 to 0.80	0.88	0.87 to 0.89	0.81	0.80 to 0.82
AUC	0.703	0.667 to 0.740	0.714	0.676 to 0.751	0.726	0.690 to 0.762
NRI, overall	Reference		0.02	–0.04 to 0.08	0.05	–0.01 to 0.10
NRI, events	Reference		–0.07		0.03	
NRI, nonevents	Reference		0.10		0.02	

Basic model (model 0): high risk group defined as FRAX score for hip fracture ≥3.0; Simple model (model 1): final model using CART with continuous variables of total hip BMD, femoral neck BMD, and age; complex model (model 2): final model using CART with continuous variables of FRAX score, femoral neck BMD, and age.

CART = classification and regression tree analysis; AUC = the area under the receiver‐operating characteristic curve; NRI = the net reclassification improvement index.

**Figure 2:**
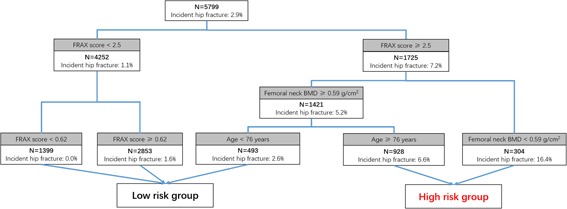
The structured risk tree for hip fracture prediction developed by CART (classification and regression tree) analysis—from model 2.

In the complex model (model 2), the FRAX score, age, and femoral neck BMD were selected as the most important predictors (The absolute cross‐validated error rate for 10‐group internal validation was approximately 22%, see Fig. 2.). The first node was above or below a FRAX score of 2.5. For those below this value, there were two additional nodes: femoral neck BMD above or below 0.59 g/cm^2^ and age above or below 76 years. Two subgroups were identified as high risk for hip fracture. Hence, 1232 (20.6%) men were identified as high risk for hip fracture and 111 (9.0%) of whom had an incident hip fracture during the 10‐year follow‐up. There were 4745 (79.4%) men identified as low risk for hip fracture; 61 (1.3%) of whom had an incident hip fracture. Compared with the low‐risk group, the HR of the high‐risk group was 8.60 (95% CI, 6.29 to 11.76; see Table 2). When using the category of FRAX score (≥3.0%) instead of the continuous one in the secondary analysis (model 4), no further factor was selected for risk discrimination at the clinical risk level of 3.0%.

The predictive performance of the basic model (model 0), the simple model by CART (model 1) and the complex model by CART (model 2) were compared for hip fracture prediction. Compared with the basic model (model 0), the high‐risk groups identified by the simple model using CART (model 1) had a lower sensitivity of 0.55 (95% CI, 0.47 to 0.62), but a higher specificity of 0.88 (95% CI, 0.87 to 0.89), and a nonsignificantly improved AUC of 0.714 (95% CI, 0.676 to 0.751). Compared with the basic model (model 0), the overall NRI was 0.02 (95% CI, –0.04 to 0.08). The performance had no statistical difference between the risk groups from the complex model developed by CART (model 2) and the basic model (model 0) (sensitivity: 0.62 versus 0.65; specificity: 0.78 versus 0.81; AUC: 0.703 versus 0.726, *p* = 0.102; overall NRI: 0.05, *p* = 0.101; Table 2). Overall, excluding men with bisphosphonate use (*N* = 515, 8.6%) had little impact on the results (data not shown).

## Discussion

In the present study, classification models based on the continuous variables of age, total hip BMD, femoral neck BMD, common clinical risk factors with or without FRAX score, and osteoporosis (defined by hip BMD *T*‐score) were developed using the method of CART for older American men. The most important discriminators selected by CART were FRAX score, hip BMD, and age. Compared with the traditional risk classifications by FRAX score at the clinical risk level of 3.0% in the United States, the classification models by CART (using hip BMD and age) performed statistically equally for hip fracture prediction. Although the FRAX category is an important clinical indicator for hip fracture risk, the simple classification by age and BMD using CART may be equally good for the estimation of risk.

Since being introduced by the WHO, the 10‐year absolute fracture risk estimated by FRAX has been used as a basis for clinical risk judgment, with an established threshold of 3.0% for hip fracture in the United States.^5^, ^25^ Though this existing practice recommendation has been widely adopted, it does not classify risk perfectly. More precise risk stratification might be needed.^26^ Tree‐structured survival analysis has been reported for American women, showing it to be a useful and reproducible procedure for the identification of meaningful prognostic subgroups based upon an individual woman's age and BMD measurements for hip fracture risk.^27^ However, no such exploration has been completed in men. No study has tested its utility within the consideration of clinical meaning. Our present study shows the hip fracture risk prediction performance of classification models developed in a CART analysis is reasonably good, and is not essentially different with FRAX categorization based on 10‐year risk of hip fracture at the clinical risk level of 3.0%.

Many tools and methods have been used to find an optimal approach for fracture prediction. Among these, FRAX earned its fame by its absolute risk‐based approach for prediction.^4^ Moreover, the use of a FRAX threshold of 3.0% provided a basis for shared decision‐making between patient and physician for hip fracture prevention in the United States.^5^, ^26^ Our present study finds that some high‐risk groups could be identified by simple measures (such as BMD and age) as illustrated by our CART results. Total hip BMD was the strongest discriminator among those factors, followed by age and femoral neck BMD. In general, like total hip BMD *T*‐score ≥–0.4 (absolute value approximately 0.89 g/cm^2^) shown here, no matter the age or other factors, the absolute hip fracture risk is relatively low in older men. This strong predictive role of total hip BMD was consistent with previous findings using traditional methods. The simple classification model (model 1) showed a clear discrimination between the high risk and the low risk on the basis of the absolute hip fracture risk of 3.0%. Its overall predictive ability was equal to the risk category of FRAX with a little lower sensitivity and a higher specificity. Although some other factors, such as previous fracture, fall history, smoking status, or osteoporosis status, are significantly associated with incident hip fracture risk, they had a minor effect in classifying risk in these analyses. This discrepancy might be because, although these factors were related with high‐fracture risk, their ability of risk classification at the clinical risk level of 3.0% was eclipsed by much more dominant risk indicators, such as older age and lower hip BMD.

The calibrated performance of FRAX was not as robust in men as in women,^28^, ^29^ which might attenuate its efficiency somewhat in clinical practice. In the present study, the optimal threshold of the FRAX score to identify the 3.0% hip fracture probability in older men was not an exact 3.0%, as the healthy economic analysis chose, but a little lower at 2.5%. However, using a FRAX score of 2.5%, together with the femoral neck BMD value of 0.59 g/cm^2^ and age value of 76 years, was not materially different in terms of risk stratification than the use of a FRAX score of 3.0%. Moreover, if using the category of FRAX score (≥3.0%), no factor was further selected for risk discrimination. This demonstrates a reasonably good performance of FRAX category at the level of 3.0% in the present study, which is no different from a model with age and BMD alone.

We have provided an example of the classification of risk groups using CART analysis for the prediction of hip fracture in MrOS; however, the reproducibility of our results may not be guaranteed in other populations of men, in women, or in other race or ethnic groups. First, the performance in classification in the present study may be impacted by overfitting, although we used a common C_p_ (equal to 0.01) to control it and used 10‐fold cross‐validation to measure this effect. In addition, we had only 172 incident hip fracture cases for analysis so the statistical power would be limited for comparison of small effect size (such as differences in discrimination statistics). Thus, the predictive value may be specific to MrOS. Second, the comparison results of predictive ability might be different in women, as the calibrated performance of FRAX was not as robust in men as in women.^28^, ^29^ Third, other studies have suggested that total hip BMD may be more strongly associated with the risk of hip fracture in men than in women,^30^ which suggests that our results may not necessarily apply to women. MrOS consists of mostly non‐Hispanic white men, so whether similar results would be seen in other race and ethnic groups is not known. MrOS is an epidemiological observational study with the possibility of a healthy volunteer bias. The current risk‐identified pattern may be specific to this more healthy population.

In addition, inherent in the CART models, only one tree is reported; there may be many nearly equal trees resulting in different absolute cutoff values in the same discriminator or different discriminators entirely that similarly classify risk.^27^ The goal of the present study was not to look for new cutoff values or algorithms for fracture risk calculation, but to show a simple way to identify the high hip fracture risk using common factors; exact cutpoints to identify those at very high risk of fracture would need to be developed in population representative samples. Finally, although high‐traumatic fractures caused by accidents were assumed to be associated with low BMD,^31^ we excluded them to reduce the possible noise induced by the potentially stochastic nature of these events. Because FRAX 10‐year estimates are for fractures of any degree of trauma, an evaluation not including them should not essentially affect the estimates.

We conclude that a simple risk categorization based on hip BMD and age demonstrated a reasonably good performance when compared with the FRAX category for hip fracture prediction at the clinical risk level of 3.0% in this cohort of older men. Although FRAX is an effective classifier of fracture risk, age and BMD together can also identify subgroups with very high and very low risk of fracture. The decision trees used in this study provide just one example of how age and BMD can be combined to determine fracture risk; future research should consider whether these simple measures perform as well in other populations.

## Disclosures

All authors state that they have no conflicts of interest.
